# Diagnostic Policies Optimization for Chronic Diseases Based on POMDP Model

**DOI:** 10.3390/healthcare10020283

**Published:** 2022-02-01

**Authors:** Wenqian Zhang, Haiyan Wang

**Affiliations:** School of Economics and Management, Southeast University, Nanjing 211189, China; wzhang@seu.edu.cn

**Keywords:** chronic diseases, partially observable Markov decision process, diagnostic threshold, diagnostic policies, policies optimization

## Abstract

During the process of disease diagnosis, overdiagnosis can lead to potential health loss and unnecessary anxiety for patients as well as increased medical costs, while underdiagnosis can result in patients not being treated on time. To deal with these problems, we construct a partially observable Markov decision process (POMDP) model of chronic diseases to study optimal diagnostic policies, which takes into account individual characteristics of patients. The objective of our model is to maximize a patient’s total expected quality-adjusted life years (QALYs). We also derive some structural properties, including the existence of the diagnostic threshold and the optimal diagnosis age for chronic diseases. The resulting optimization is applied to the management of coronary heart disease (CHD). Based on clinical data, we validate our model, demonstrate how the quantitative tool can provide actionable insights for physicians and decision makers in health-related fields, and compare optimal policies with actual clinical decisions. The results indicate that the diagnostic threshold first decreases and then increases as the patient’s age increases, which contradicts the intuitive non-decreasing thresholds. Moreover, diagnostic thresholds were higher for women than for men, especially at younger ages.

## 1. Introduction

Chronic diseases remain one of the major public health problems in the world, imposing a high cost burden on healthcare systems and reducing the quality of life of patients [1,2,3]. About half of all adults have one or more chronic diseases, which are responsible for 70% of deaths in the United States and account for more than 85% of total healthcare expenses [4]. In China, chronic diseases contribute 86.6% to total deaths, and the burden accounted for 70% of the total medical cost in 2017 [5]. Given that the population is increasing and aging, especially due to the impact of COVID-19, people are more concerned about their health. Therefore, how to take effective intervention strategies early enough to be effectively diagnosed is of paramount importance [6].

There are many advanced medical examinations for screening and diagnosing chronic diseases, including non-invasive and invasive tests, which makes it possible to make personalized diagnostic policies based on individual characteristics to detect chronic diseases early [7]. Non-invasive examinations have important value for early screening of chronic diseases. When a patient undergoes a non-invasive test for basic screening, a physician will decide whether he needs further invasive tests based on the screening results, such as coronary angiography (CAG), prostate biopsy, or breast biopsy. Such policies for invasive tests made following basic screening are called diagnostic policies [8].

The latest screening program of the U.S. Preventive Services Task Force emphasizes that the purpose of screening should be to maximize the life of the patient while minimizing the damage to the body [9]. When diagnosing chronic diseases for patients, a trade-off is required between the long-term benefits of early detection on the one hand and the short-term side effects of invasive tests and the long-term side effects of subsequent treatment on the other hand. Meanwhile, overdiagnosis (unnecessary examinations) cause patients anxiety and increases personal and national medical costs, whereas underdiagnosis (lack of tests) means patients may not be treated in time [10,11,12,13]. Thus, the underdiagnosis–overdiagnosis trade-off must be confronted for chronic diseases, which serves as our motivation.

Therefore, we address the following research questions about chronic disease diagnosis based on differences in specificity, sensitivity, and associated side effects of the above different examinations:

1. Whether and when should a physician advise an individual patient to take an invasive test based on basic screening results via non-invasive tests?
2. How should physicians and decision makers in health-related fields make diagnostic policies for populations with various individual characteristics?

Our motivating example is CAG services for coronary heart disease (CHD) diagnosis. The risk of CHD is predominant among all diseases, and about one in three adults has CHD [14]. Furthermore, patients with CHD are at greater risk of death with COVID-19 than patients with other chronic diseases [15]. Common diagnostic methods include electrocardiogram (ECG), computed tomography (CT), and CAG. ECG and CT are convenient and non-invasive and are often used as a basic screening tool to detect CHD at its early stages, yet they are prone to misdiagnosis and missed diagnosis due to low specificity and sensitivity; CAG is invasive and expensive as well and entails related side effects.

The results of CAG in some patients, however, are normal in the clinic. Currently, some studies show that the proportion of normal examination results is about 16.9–31.1% in China [16]. Such overdiagnosis is not unique to China: For instance, data reports from 663 hospitals in the American College of Cardiology National Cardiovascular Data Registry showed that 62.4% of patients had no obstructive coronary artery disease in 2010 [17,18]. Hatzidakis et al. [19] indicated that no lesions or nonsignificant lesions were found (47.4% of patients). This implies that unnecessary tests can result in excessive diagnosis and additional medical costs. Furthermore, individual characteristics have a significant impact on the diagnostic rate of CAG [20,21,22,23].

To do so, we constructed a partially observable Markov decision process (POMDP) model for quantitative trade-offs to optimize diagnostic policies for patients with suspected chronic diseases, which can help governments to optimize the allocation of medical resources. Our objective is to maximize a patient’s total expected quality-adjusted life years (QALYs). Moreover, combined with clinical data, we evaluated the performance of optimal policies on CAG diagnosis and compared them with physicians’ decisions in clinical practice.

Different from the existing studies, we studied optimal diagnostic policies and analyzed the impact of individual characteristics of patients on them. To the best of our knowledge, this is the first study from two different perspectives (i.e., individual perspectives and population perspectives) that determines optimal policies for clinical diagnosis based on the POMDP model. The updating of belief state makes our model more consistent with the imperfect nature of basic screening and partially observable health conditions in chronic diseases, which is the difference between our POMDP model and other Markov models in the related literature.

The remainder of our study is organized as follows. In Section 2, we review the relevant literature about chronic diseases management and POMDP in medical decision-making, especially about CHD. We characterize the problem of the diagnostic decision in Section 3. In Section 4, we propose the POMDP model and describe the optimal equation as well as some properties. In Section 5, a case study of CHD is presented to illustrate the applicability of the proposed model. The discussion and conclusion are revealed in Section 6 and Section 7, respectively.

## 2. Literature Review

This paper is related to chronic diseases management and POMDP applications in medical decision-making. We review the related literature as follows, and then point out the differences between our study and existing literature.

### 2.1. Chronic Diseases Management

Research on chronic disease policies has been primarily conducted in terms of three aspects: screening, treatment, and diagnosis. Many studies mainly focus on cost–benefit analysis of screening policies and treatment policies about chronic diseases, such as hypertension [2], breast cancer [8,24], liver cancer [25], colon cancer [26], and glaucoma [3] based on Markov models.

Disease diagnosis plays a critical role in designating screening and treatment policies [27]. In making diagnosis decisions, physicians must consider the trade-off between detecting diseases early and sparing healthy people unnecessary procedures [28]. There are many studies on diagnostic policies of prostate cancer [29] and breast cancer [8,30], which demonstrate that the threshold to biopsy increases with age, implying less potential benefit of a biopsy in older ages.

Regarding decision-making on CHD, while there have been many studies that focus on the optimal screening policies [31,32] and treatment policies [33,34,35,36], there have been relatively few studies that focus on improving CHD diagnosis based on quantitative trade-offs. On the other hand, it is worth noting that the heterogeneity of patients is of paramount importance in making decisions [2,29,37]. Due to the heterogeneity in the patient population with CHD, the impact of treatment may vary among subgroups of the overall population in terms of resource use, health benefits, and cost-effectiveness [38].

### 2.2. POMDP Applications in Healthcare

In healthcare, Markov models are extensively used for decision-making [39]. The use of POMDP in the health context is rather recent, which has attracted much attention within operations management.

As we all know, the POMDP model is a generalization of the Markov decision process (MDP), which is the most popular stochastic sequential decision process in reinforcement learning. Because patients’ condition evolves with time, the effects of diagnosis can be accessed via POMDP, and it is not assumed that the state of the system is fully observable, which is consistent with reality and makes it suitable to study chronic diseases.

POMDP has been used to study optimal disease decisions by considering different factors in the literature, such as individual risk factors [37], heterogeneity of adherence rates [40,41], and limited mammography resources [42]. In addition, some studies determine the best time for drug susceptibility testing [43] and analyze the telemedicine triage system [44].

Among the aforementioned literature, Zhang et al. [29] is the study most related to our study. They assumed that the biopsy rate was fixed at different ages. Notwithstanding the relevance, our paper differs from it in three aspects. First, we not only consider the impact of age and gender on the prevalence and deterioration rate, but also analyze dynamic changes under various individual characteristics in the model. Second, we specifically prove some unique structural properties including the optimal diagnosis time for a certain patient with CHD and the belief threshold of diagnostic policies for different populations, while they focused on population-based policies. Third, due to the specificity of CHD and its examinations, our model differs from other literature. For instance, patients with suspected CHD will have health loss, and the transfer of health status is different from other diseases. In our study, the threshold first decreases and then increases as the patient’s age increases, which is different from the non-decreasing thresholds in other studies. Finally, combined with clinical data, we evaluate the performance of optimal policies on CAG diagnosis and compare them with physicians’ decisions in clinical practice.

## 3. Problem Definition

**Figure 1:**
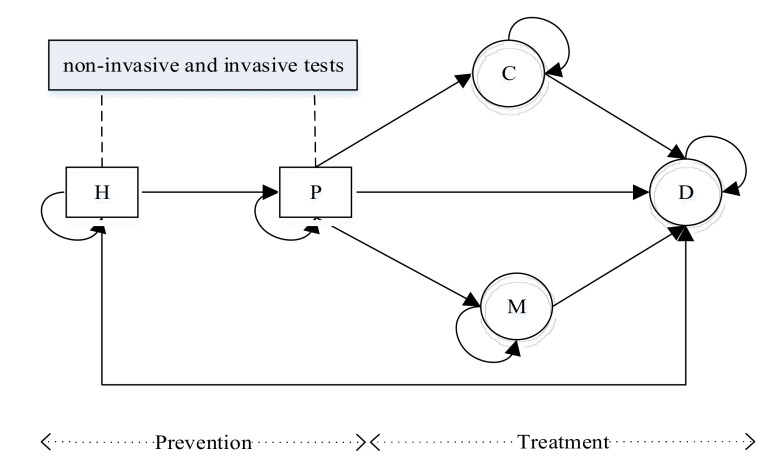
State transition diagram.

In our model, a patient is in one of five health states including health (H), pre-clinical state (P), clinical state (C), deterioration (M), and death (D), as shown in Figure 1. The state P indicates that the disease is present but not detected, while the state C indicates that the disease is detected through an invasive test. In addition, the state M indicates that the patient’s condition has deteriorated. The states in our model are associated with disease incidence and progression as well as patient mortality.

If the patient is diagnosed with diseases, his health status will be transferred to state C. In this state, he can be treated with drugs and possibly surgery. For example, if patients are diagnosed with CHD, many of them may be treated with drugs, while some of these patients with severe conditions may undergo PCI or CABG. This means they will exit the diagnosis system and start treatment, and hence we assume a patient in a clinical state (C) will not transition to a deteriorating state (M).

Patients in states H and P can be screened by non-invasive tests such as ECG and CT. Due to the limitation and the inaccuracy of these tests, they cannot confirm whether the patient has diseases, which implies that the patient’s condition is partially observable. Because of this feature, we use a probability distribution to represent the belief on true states and update the belief states in a Bayesian manner based on test results.

**Figure 2:**
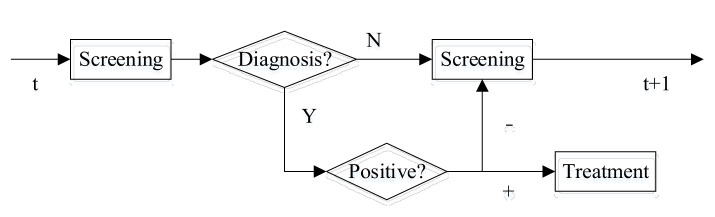
Decision-making process of diagnosis.

A diagnostic process is illustrated in Figure 2. When a patient undergoes non-invasive tests, a physician assesses screening results and makes a decision from the two alternatives: wait or administer invasive tests. That is, the physician decides whether to administer an invasive test for the patient based on the results of the basic screening and the value of updated risk. If the decision is to wait, the patient will continue to perform non-invasive tests and make a decision for the same problem in the next year; if the decision is diagnosis via an invasive test, the patient will have two results after the examination: (1) health, the patient will stay in the current state and perform non-invasive tests at next decision epoch; (2) diagnosed with diseases, the patient’s health status will be transferred to state C, which implies that he will exit the diagnosis system and start treatment. Patients diagnosed with diseases will be treated, implying that there are long-term side effects of subsequent treatment.

In our model, QALYs is chosen as the criteria since it is extensively used in evaluating the benefits of patients in the aforementioned literature. By capturing the decrements in the quality of life due to side effects of related events such as tests and treatment, it can consider both quality and quantity of life under different health conditions. Specifically, QALYs is normalized to 1 with perfect health for a patient in a full year, and 0 for death [45].

We describe various assumptions made throughout our model. Due to the high accuracy of invasive tests, patients will leave this diagnostic system immediately once they take an invasive test, which is consistent with previous medical decision-making studies [29,37,40,41,42], and hence we assume a patient in state C will not transition to state M. On the other hand, once a patient’s health status has deteriorated, we assume that he will exit the diagnosis system because our model focuses on preventive diagnosis rather than treatment, and therefore we assume a patient in state M will not transition to state C. In addition, because it is not reported in the relevant literature that non-invasive tests will reduce QALYs of patients, we assume that there is no QALYs decrement associated with basic screening. In recent years, many patients have participated in physical examinations every year in hopes of detecting diseases in China. In light of this case, we assume that patients receive basic screening annually. Lastly, we assume that patients fully comply with physicians’ diagnosis recommendations, since physicians are often altruistic and act in the best interest of patients.

Therefore, on the one hand, from the patient’s perspective the optimal diagnosis age for a certain patient should be determined based on his basic screening results, which means that this patient should be recommended to diagnose via invasive tests in this time, namely optimal diagnosis time. On the other hand, from the perspective of physicians and the government as well as decision makers, we analyze optimal diagnostic policies, denoted by the belief threshold between administering invasive tests and waiting, for populations with various individual characteristics. Our objective is to maximize patient health benefits, thereby efficiently balancing underdiagnosis and overdiagnosis.

## 4. Mathematical Model

As mentioned earlier, we developed a POMDP model to maximize a patient’s total expected QALYs for optimizing policies in chronic diseases diagnosis, which can characterize the diagnosis progress. The model combines the health states of patients with decisions and updates their belief states (i.e., the probability of being in various disease stages) at each decision epoch according to basic screening results. Next, we detail our POMDP model.

The decision epochs in our model are denoted by $t\in T=\left\{40,41,42,\ldots ,100\right\}$. That is, the starting age of screening is 40 years old, and the ending age is 100 years old. Assume that each screening is conducted at the beginning of each year.

At period t, health states of patients are denoted by $s_{t}\in S=\left\{H,P,C,M,D\right\}$. H and P are partially observable, whereas the other states are completely observable. Patients have a basic screening via non-invasive tests once a year, so the state transition interval is one year.

The action is taken by a patient aged t, denoted by $a_{t}\in A=\left\{G,W\right\}$. The action space A consists of two decisions: diagnosis via an invasive test (G) or wait (W) until the next decision epoch. Specifically, if the health state of the patient $s_{t}\in \left\{H,P\right\}$ at the start of year is t, he will undergo basic screening, and then the physician advises whether he will be subjected to invasive testing ($a_{t}=G$) or not ($a_{t}=W$) based on basic screening results in that year.

### 4.1. Observation States and Observation Probabilities

The health states of patients can be obtained through observable health statuses and basic screening results. Due to the imperfect nature of basic screening, states H and P are partially observable. However, some observation information can be obtained that can be used to make further decisions. The observation space is defined as $O=\left\{PH,PP,C,M,D\right\}$. We use PH to denote an observed health state by results, and PP to denote an observed CHD present but not diagnosed state according to results. All other states are fully observable.

We let $l_{t}$ denote the observation when the patient is in a true state $s_{t}$ at time t, and use $q_{t}\left(l_{t}\left|s_{t}\right.\right)$ to denote the probability of observing $l_{t}$. We define the observation probability matrix as $Q_{t}\left(l_{t}\left|s_{t}\right.\right)$. Note that $Q_{t}\left(l_{t}\left|s_{t}\right.\right)$ is independent of the actions because actions are only taken for the patient in states $s_{t}\in \left\{H,P\right\}$ and these actions do not affect basic screening results. If he is in health state $s_{t}\in \left\{C,M,D\right\}$, it implies that the disease has been diagnosed and thus no invasive test is needed and the observation on the state is deterministic and reflects the true state, i.e., $q_{t}\left(l_{t}\left|s_{t}\right.\right)=1$ for $l_{t}\in \left\{C,M,D\right\}$ and $q_{t}\left(l_{t}\left|s_{t}\right.\right)=0$ for $l_{t}\in \left\{PH,PP\right\}$, e.g., $q_{t}\left(C\left|C\right.\right)=1$. With a certain probability (specificity), the result of a healthy patient will be negative; with a certain probability (sensitivity), the result of a patient with CHD will be positive. We let $p_{1}$ and $p_{2}$ denote the specificity and the sensitivity of basic screening, respectively, and then

(1) $$Q_{t}\left(l_{t}\left|s_{t}\right.\right)=\left(\begin{array}{cc} p_{1} & 1-p_{1}\\ 1-p_{2} & p_{2} \end{array}\right),$$

where rows refer to states H and P and columns refer to observed states PH and PP.

### 4.2. Transition Probabilities

We let $p_{t}\left(s_{t+1}\left|s_{t},a_{t}\right.\right)$ denote health state transition probability, i.e., the probability of the patient in state $s_{t+1}$ at age t+1, given that he was in state $s_{t}$ and took action $a_{t}$ at age t. Taking CHD as an example, if $a_{t}=W$, the patient’s CHD-related health state evolves naturally. CHD is caused by a narrowing of the blood vessels in the heart resulting in inadequate blood supply. Even if patients take drugs for a long time, it can only slow down the progression of CHD, not cure it. The patient’s health condition will not be reversed but will only remain the same or deteriorate. There are some parameters used to construct the state transition probability matrices in our model. We let $i_{t}$ and $e_{t}$ be the annual incidence of CHD and the probability of health deterioration which are age-specific, and $d_{1}$ and $d_{2}$ be the annual mortality from CHD and other reasons, respectively.

If $a_{t}=G$, CAG test changes the course of CHD natural disease progression in that it triggers treatment at a clinical state. We assume that the CAG test can diagnose CHD and identify the patient’s state with certainty.

For $s_{t}\in \left\{H,P\right\}$, if the patient is diagnosed with certainty by CAG ($a_{t}=G$), the patient will be transferred to state C with certainty and the probability of transitioning to any other states is 0.

For $s_{t}\in \left\{C,M,D\right\}$, no CAG is needed and $p_{t}\left(s_{t+1}\left|s_{t},a_{t}\right.\right)$ does not depend on $a_{t}$. Hence, the patient’s health state evolves naturally, which can be modeled by setting $p_{t}\left(s_{t+1}\left|s_{t},W\right.\right)=p_{t}\left(s_{t+1}\left|s_{t},G\right.\right)$.

### 4.3. Belief States and Belief Update

We denote a belief state (or belief vector) to be $b_{t}=\left(b_{t}\left(H\right),b_{t}\left(P\right),b_{t}\left(C\right),b_{t}\left(M\right),b_{t}\left(D\right)\right)$, where $b_{t}$ is the probability with which a person is in one of the five health states at epoch t. We let B be the set containing all possible belief states, i.e., $b_{t}\in B\equiv \left\{b_{t}\in {\mathbb{R}}^{5}\left|\sum_{s\in S}b_{t}\left(s\right)\right.=1,0\leq b_{t}\left(s\right)\leq 1,s\in S\right\}$, where ℝ denotes the set of real numbers. Note that, if a patient has an observation $l_{t}\in \left\{C,M,D\right\}$, his belief state is $b_{t}\left(s_{t}\right)=1$ for $s_{t}\in \left\{C,M,D\right\}$. This indicates that our five-state POMDP model includes two partially observable states (H and P) and three absorbing states (C, M, D). For a patient without a positive result of invasive tests, his belief state can be represented as $b_{t}=\left(1-b_{t}\left(P\right),b_{t}\left(P\right),0,0,0\right)$.

If $l_{t}\in \left\{PH,PP\right\}$, we apply Bayesian updating for the patient’s belief state $b_{t}$ based on $l_{t}$ and $a_{t}$, which can be used to revise the belief state $b_{t+1}$. Based on [29,41,42], the updated belief can be obtained by the following transformation

(2) $$b_{t+1}\left(s_{t+1}\right)=\frac{q_{t+1}\left(l_{t+1}\left|s_{t+1}\right.\right)\sum_{s_{t}\in S}p_{t}\left(s_{t+1}\left|s_{t},a_{t}\right.\right)b_{t}\left(s_{t}\right)}{\sum_{{s^{\prime }}_{t+1}\in S}q_{t+1}\left(l_{t+1}\left|{s^{\prime }}_{t+1}\right.\right)\sum_{s_{t}\in S}p_{t}\left({s^{\prime }}_{t+1}\left|s_{t},a_{t}\right.\right)b_{t}\left(s_{t}\right)};$$

If $l_{t}\in \left\{C,M,D\right\}$, no belief needs to be updated because the true disease state can be observed.

### 4.4. Rewards

We define the rewards which incorporate the expected future longevity and side effects associated with tests. Let ${\bar{r}}_{t}\left(s_{t},a_{t}\right)$ be the immediate reward (measured in QALYs) when the patient’s true health state is $s_{t}$ and action $a_{t}$ is selected at time t, and hence the belief state expected immediate reward is computed by $r_{t}\left(b_{t},a_{t}\right)=\sum_{s_{t}\in S}b_{t}\left(s_{t}\right){\bar{r}}_{t}\left(s_{t},a_{t}\right)$. In addition, let $p_{3}$ be the diagnostic rate of invasive tests for patients, i.e., the probability of detecting the disease through an invasive test (such as CAG) in state P, μ be the expected loss associated with a single invasive test, and let α, β, and γ denote the annual expected loss of QALYs in states P, C, and M, respectively. We assume that all parameters and immediate rewards have values in $\left[0,1\right]$. Meanwhile, we assume $\mu +\alpha \left(1-p_{3}\right)+\beta p_{3}<1$ and α≤β≤γ. Immediate rewards are provided in Table 1.

### 4.5. Optimality Equation

Our objective is to determine optimal diagnostic policies for chronic diseases, which maximizes the patient’s total expected discounted QALYs. The optimal value function of the model can be written as

(3) $$v_{t}\left(b_{t}\right)=\underset{a_{t}\in \left\{G,W\right\}}{\max}\left\{r_{t}\left(b_{t},a_{t}\right)+\lambda \sum_{l_{t+1}\in O}v_{t+1}\left(b_{t+1}\right)p_{t}(l_{t+1}\left|b_{t},a_{t}\right.)\right\},\forall b_{t}\in B,$$

where $p_{t}(l_{t+1}\left|b_{t},a_{t}\right.)=\sum_{s_{t+1}\in S}^{}q_{t+1}(l_{t+1}\left|s_{t+1})\sum_{s_{t}\in S}^{}p_{t}(s_{t+1}\left|s_{t},a_{t}\right.\right.)b_{t}(s_{t})$ and $\lambda \in \left[0,1\right]$ denotes the annual discount factor. In the optimality equation, the first component represents the expected immediate reward of a patient at decision epoch t, and the other represents the total expected future rewards. Thus, the diagnostic policy depends not only on current immediate rewards but also on future rewards. The larger the discount factor, the more important future rewards.

As previously assumed, patients will leave our diagnostic system immediately once they take an invasive test, and hence the optimal diagnostic policy can be viewed as a partially observable optimal stopping time problem. Let $v_{t}\left(b_{t},W\right)$ and $v_{t}\left(b_{t},G\right)$ be the total expected discounted QALYs for actions W and G in belief state $b_{t}$, respectively. At each decision epoch, the physician will choose between the expected reward associated with invasive tests, $v_{t}\left(b_{t},G\right)$, or deferral of the decision to invasive tests to the next decision epoch, $v_{t}\left(b_{t},W\right)$. Thus, the optimality equation can be rewritten as

(4) $$v_{t}\left(b_{t}\right)=\max\left\{v_{t}\left(b_{t},W\right),v_{t}\left(b_{t},G\right)\right\},\forall t,\forall b_{t}\in B.$$

Let ${\bar{R}}_{t}\left(s\right)$ denote the expected discounted future reward given the patient is never referred for invasive tests (i.e., $a_{t}=W$), and is in state $s_{t}$, in age t. On the contrary, $R_{t}\left(s\right)$ is the expected discounted future reward given $a_{t}=G$ in decision epoch t. Lastly, the difference between a patient’s health rewards under two strategies (wait and diagnosis) can be calculated as:

(5) $$\Delta QALYs=v_{t}\left(b_{t},G\right)-v_{t}\left(b_{t},W\right).$$

### 4.6. Structural Properties

In this section, we discuss the structure of our POMDP and prove relevant propositions to provide insights to decision makers on optimal diagnostic policies of chronic diseases. POMDPs are computationally difficult to solve. However, it was shown by Smallwood and Sondik (1973) [46] that the value functions for POMDPs are finite, piecewise linear, and convex, and POMDPs can be converted into an equivalent completely observable Markov decision process on the continuous belief states [9,29,47,48], which greatly simplifies the solution of our model.

According to previous assumptions, we first analyze optimal diagnostic decisions by measuring the difference of health rewards under different strategies. Next, we demonstrate that optimal policies of chronic diseases diagnosis follow a control-limit type policy with respect to a belief threshold. The proofs of all results in this section are presented in Appendix A.

**Proposition** **1.** *Under the assumptions above, for any*∀t∈T*, we have*$\frac{d\Delta QALYs}{db_{t}\left(P\right)}\geq 0$.

Proposition (1) presents that ΔQALYs increases with $b_{t}\left(P\right)$ (the probability of having chronic diseases) at each period t. As a result, compared to waiting, patients with high risk tend to diagnose by invasive tests. This suggests that an increased risk of chronic diseases naturally increases the future reward of diagnosis and reduces the relative effect from side effects of an invasive test and subsequent treatment on patients.

**Proposition 2.**  *There exists an optimal diagnosis time*$t^{*}$for the patient with the probability of having chronic diseases $b_{t}\left(P\right)$, and the optimal time is uniquely determined by the following equation

(6) $$t^{*}\in \arg\max\left(v_{t}\left(b_{t},G\right)-v_{t}\left(b_{t},W\right)\right).$$

Proposition (2) indicates that there is the maximum incremental rewards in QALYs of chronic diseases diagnosis at time $t^{*}$ between two diagnostic decisions in belief state $b_{t}\left(P\right)$, and the patient’s optimal policy is diagnosis by an invasive test, i.e., $a_{t}^{*}=G$. From the patient’s perspective, Proposition (2) shows how a physician determines the optimal time for a single patient in belief state $b_{t}\left(P\right)$ based on ΔQALYs. Next, Proposition (3) will make optimal diagnostic policies for populations from the perspective of physicians and governments.

**Proposition** **3.** *There exists a threshold* $b_{t}^{*}\left(P\right)$ *such that*

*1.*  *If* $b_{t}\left(P\right)\leq b_{t}^{*}\left(P\right)$*, the optimal policy is wait, i.e.,* $a_{t}^{*}\left(b_{t}\right)=W$;
*2.*  *If* $b_{t}\left(P\right)>b_{t}^{*}\left(P\right)$*, the optimal policy is diagnosis, i.e.,* $a_{t}^{*}\left(b_{t}\right)=G$;

*where the threshold equals*

(7) $$b_{t}^{*}\left(P\right)=\frac{\mu }{R_{t}\left(P\right)-{\bar{R}}_{t}\left(P\right)+\mu }.$$

Proposition (3) states that optimal diagnostic policies exhibit a threshold structure. This implies that $b_{t}^{*}\left(P\right)$ is the maximum probability that the patient who chooses to wait is in state P. When a patient’s belief state $b_{t}\left(P\right)$ is lower than this threshold, the optimal policy is wait, while when $b_{t}\left(P\right)$ is higher than this threshold, the optimal policy is performing an invasive test.

Propositions (2) and (3) provide insights for physicians and governments as well as policymakers to make diagnostic decisions more effectively. Specifically, from the patient’s perspective, Proposition (2) indicates that a given patient should be recommended to diagnose in the optimal diagnosis time based on his basic screening results. In addition, from the government and policy makers’ perspective, Proposition (3) shows that optimal diagnostic policies are denoted by the belief threshold between diagnosis and waiting for different populations with various individual characteristics, thereby efficiently improving health and balancing between overdiagnosis and underdiagnosis. For example, if available resources are restricted, such as essential medical supplies and well-trained personnel, diagnostic policies can be made for patients with different disease probabilities in an optimized manner to effectively use resources and improve overall QALYs.

## 5. Case Study

In this section, we perform numerical analyses on CHD that investigate how patient characteristics and model parameters may impact diagnostic policies by CAG as well as health rewards of patients. The primary goal of this research is to verify the feasibility of the proposed model and provide relevant insights obtained from the results to physicians and decision makers in health-related fields for diagnosing CHD, effectively.

First, we summarize parameters from the available clinical data and present optimal policies in the basic model. Second, to achieve a more personalized diagnostic decision, we consider the influence of individual characteristics on optimal policies. Third, we evaluate the performance of optimal diagnostic policies on CAG diagnosis and compare them with physicians’ decisions in clinical practice. Last, we use sensitivity analysis to evaluate the influence of changes in each of the model parameters to identify which parameters most significantly affect optimal diagnostic policies.

### 5.1. Parameter Estimation

Our clinical data were obtained from a dataset of CAG examinations from a large general hospital in Kaifeng, Henan Province, collected between January and December 2019. The data consists of 1291 patients. Because we focused on primary diagnosis, we removed unrelated records, which included 399 patients who were in states of percutaneous coronary intervention (PCI) or coronary artery bypass grafting (CABG), and 59 patients with acute myocardial infarction. Finally, our data set consisted of 833 effective records. The results of data statistics showed that 495 patients did not suffer from CHD. Therefore, we assumed that the average diagnostic rate of CAG was 41% in the basic model. Table 2 summarizes the above parameters used to construct the transition probability and the rewards in our model, and all parameters are nonnegative and not greater than 1 by definition.

### 5.2. Baseline Analysis

**Figure 3:**
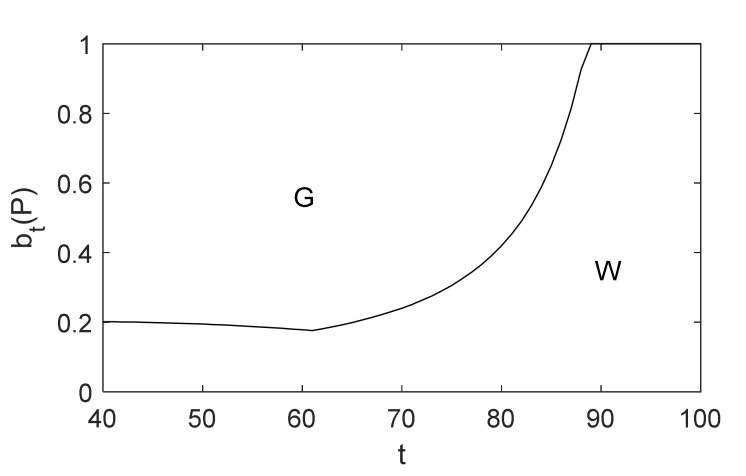
Optimal policies for diagnosis.

We discuss some interesting observations and general insights under the baseline scenario that can be drawn below. $b_{t}\left(p\right)$ at age t=40 is 0.01, which is based on the annual incidence of CHD for ages 40–60, as reported by the Center for Health Statistics and Information [49]. The belief threshold between diagnosis and waiting in the basic model is illustrated in Figure 3. Obviously, it can be seen that optimal policies have the following properties.

First, there is a threshold for optimal policies. Second, the change in the optimal threshold will be related to the age of the patient. Third, the threshold is higher for younger patients. This is because when a patient is at a young age, the health loss of undergoing a CAG is generally greater than the health loss of waiting. Fourth, there is a termination age for diagnostic decisions. If the patient is over age 88, namely the termination age, it will not be recommended to diagnose.

**Figure 4:**
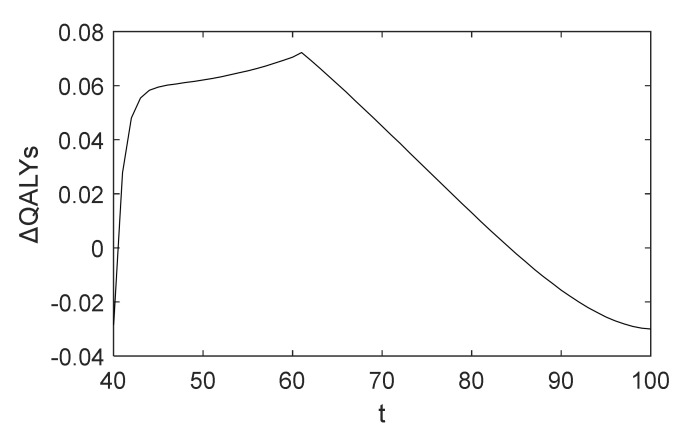
ΔQALYs in baseline analysis.

It can be seen from Figure 4 that not everyone benefits from CAG. When the patient is between age 41 and 84, the benefit of undergoing a CAG is greater than that of waiting. Especially for patients between age 43 and 66, the health rewards are significantly higher given the decision to diagnose, and the patient aged 61 who receives a CAG has the greatest health rewards. Furthermore, if patients are older than 84 years, taking CAG tests results in a decrease of their rewards, for which physicians currently hesitate to recommend patients receive a CAG for diagnosing CHD. The change is intuitive because with the increasing probability of death from other causes such as patients’ age, their remaining lifetime QALYs following treatment will be lower, which is similar to the related literature and guidelines [8,40]. Therefore, the government and decision makers in health-related fields should pay attention to patients between age 43 and 66 to promote early screening and diagnosis of CHD to reduce health loss and medical costs relating to CHD.

Unlike chronic diseases studied in other literature, we can find interesting results from Figure 3 and Figure 4. If the patient is under the age of 41, the side effects of undergoing a CAG are higher than that of waiting. Note that in this case patients will not be recommended for diagnosis, which is consistent with low adherence rates of younger patients receiving a CAG in reality. We can explain this as follows. First, because CAG is an invasive examination, it has potential risks and side effects such as bleeding or hematoma at the puncture site and arrhythmia. Secondly, CAG has proven to have a significant role in measuring the degree of vascular stenosis, and patients may be treated by PCI or CABG during the diagnosis process by performing a CAG, which is another important role. In clinical practice, a patient does not need to be treated if the degree is less than 50%. Finally, because of the lower prevalence of CHD for patients at a young age compared with other diseases, they are relatively less likely to receive treatment after diagnosis, and hence their threshold is higher. This is different from the optimal screening policies proposed in other literature, which recommend more aggressive screening for younger patients.

### 5.3. Optimal Policies Considering Individual Characteristics

In this section, in order to investigate the impact of individual characteristics on optimal policies, we updated the diagnostic rate of CAG based on the dataset described above, as shown in Table 3. The updated diagnostic rates are disaggregated according to different ages and genders.

**Figure 5:**
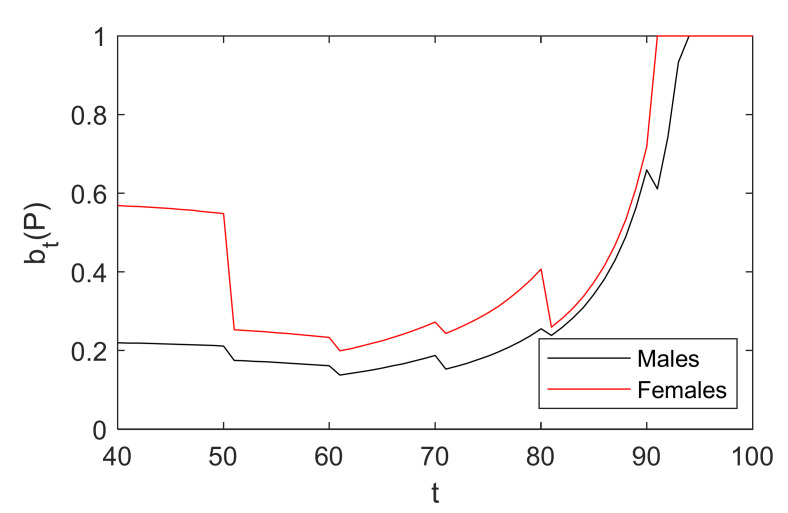
The threshold affected by individual characteristics.

As shown in Figure 5, optimal diagnostic policies vary with individual characteristics of patients; that is, the belief threshold and the termination time are different with various characteristics. In general, as the patient’s age increases, the threshold first decreases and then increases, which is different from non-decreasing thresholds in the age seen in other literature. Furthermore, females have a higher diagnostic threshold than males, especially at a younger age, and the termination time for diagnosis becomes smaller for female patients. Thus, at such low belief levels, aggressive policies would lead to many false positives and unnecessary CAG tests, and the diagnosis rate would not increase significantly, which in turn would lead to a reduction in QALY.

**Figure 6:**
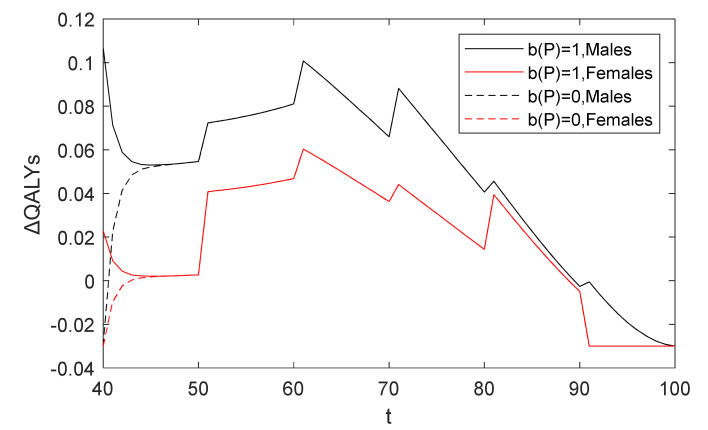
ΔQALYs affected by individual characteristics.

For evaluating the difference of health rewards, Figure 6 compares the influence of individual characteristics on the improvements in the QALY criterion under two diagnostic policies. Obviously, we find that ΔQALYs of males undergoing diagnosis is higher than that of females, which implies that having aggressive diagnosing between the ages of 50 and 75 is especially critical for males. However, the benefits for females are less because the efficacy of the screening test is lower for them. For example, the improvement between two strategies of a male aged 61 is 0.1008 QALYs, and hence a man has 0.0405 QALYs more than a woman of the same age. In addition, the diagnostic benefits of young patients may be negative due to the side effects of examinations and the low probability of having CHD, implying that the optimal policy is to wait in this case.

Therefore, physicians and decision-makers in health-related fields should focus on diagnosing patients with a high probability of having CHD via CAG to avoid underdiagnosis and delays in treatment, especially between the ages of 50 and 75. At the same time, unnecessary testing of patients with a low probability, especially young females, could be reduced, which would avoid overdiagnosis. This is in line with diagnostic guidelines and the literature [22,54,55]. It should be noted that in clinical practice, however, physicians need to consider other factors comprehensively when deciding whether a patient should go for further testing to avoid delays in diagnosis and treatment.

### 5.4. Comparison with Other Policies

We measured the total health rewards of CHD diagnosis by estimating how much the total expected QALYs for a 40-year-old patient improves when the optimal policy is adopted as opposed to other policies.

Table 4 shows that the improvement and percentage improvement in QALYs between optimal decisions and decisions in clinical practice. Based on our experiments, we found that the optimal strategies we proposed can result in higher QALY gains for patients than others. For example, the optimal policy for male patients in $b_{40}\left(P\right)=1$ is diagnosis by CAG. Compared with the decision to wait, the incremental benefit of the optimal policy is 0.1067 QALYs per person for the male population. As a result, males in a higher belief state get more rewards when taking the optimal policy than when taking others.

Note that optimal policies are different for patients of different genders. When $b_{40}\left(P\right)$ is equal to 0.5, male patients are expected to have up to 0.177% more QALYs by following the optimal policy rather than waiting, while female patients should be advised to wait. We focused on two different perspectives, i.e., individual patients and populations with various individual characteristics. Thus, the improvement in quality-adjusted life years of an individual patient is not obvious, whereas the health benefits at the population level will increase significantly as the benefits of each patient increase.

### 5.5. Sensitivity Analysis

**Figure 7:**
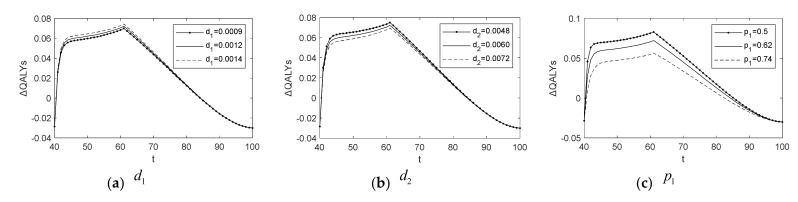

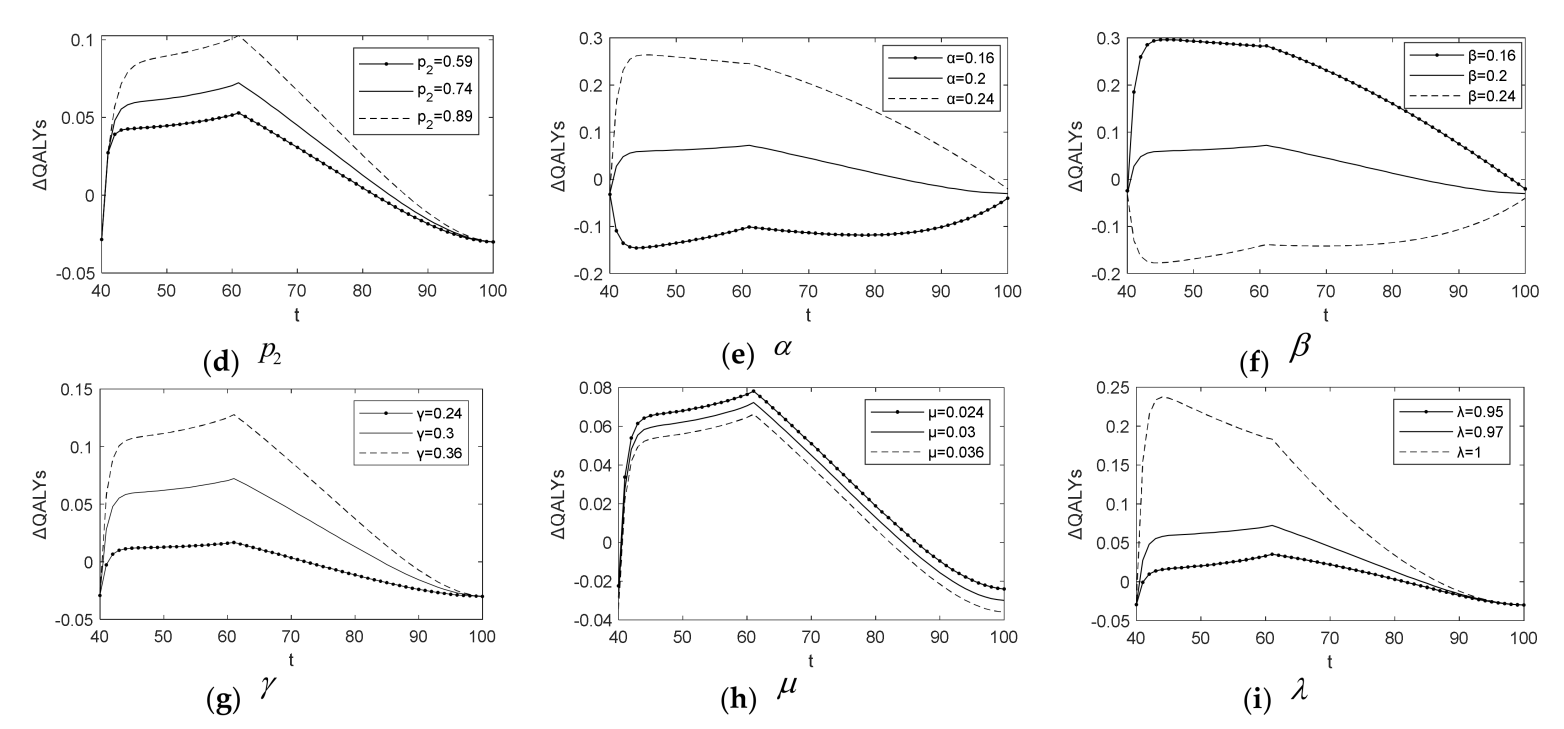
Sensitivity analysis for parameters.

Because some parameters in our model are subject to variation due to differences in actual conditions, patient preferences, and physiology, we conducted sensitivity analysis for these parameters in Figure 7. The variation range of these parameters is based on relevant literature or ±20% of the basic values [26,29].

The optimal policy is particularly sensitive to the annual loss of QALYs per person (namely α, β, and γ) in states P, C, and M, respectively, as illustrated in Figure 7e,g. In Figure 7c,d, optimal policies are sensitive to the change in specificity $p_{1}$ and sensitivity $p_{2}$ of basic screening. In addition, Figure 7i is the sensitivity analysis of the discount factor, λ. As illustrated in Figure 7a,b,h, we observe that ΔQALYs is insensitive to changes in $d_{1}$, $d_{2}$, and μ.

The above sensitivity analysis shows that it is crucial to assess patients’ health loss under each of the states when studying the diagnosis of chronic diseases. Therefore, model parameters are well estimated, which can help effectively optimize diagnostic policies based on various personal characteristics. Furthermore, improving the sensitivity of basic screening and reducing the side effects of CAG and subsequent treatment can effectively improve health rewards for undergoing a CAG, which implies that patients will be willing to diagnose.

## 6. Discussion

From the patient’s perspective, there exists an optimal diagnosis time based on basic screening results via non-invasive tests. When patients are in the recommended age, they will be encouraged to diagnose via invasive tests for maximizing their health rewards.

From the perspective of physicians and the government as well as decision makers, optimal policies of chronic disease diagnosis follow a control-limit type policy with respect to a belief threshold for different populations with various individual characteristics. This can lead to better resource utilization for healthcare providers and governments. Unlike most studies in the literature, due to the specificity of CHD and its examinations, the diagnostic threshold first decreases and then increases as the patient’s age increases. In addition, not everyone benefits from non-invasive tests, and we were able to identify patients that could benefit from tests through this model.

Therefore, physicians should focus on the diagnosis of males with a high probability of having CHD to efficiently avoid underdiagnosis and a delay in treatment, particularly between the ages of 50 and 75. In contrast, unnecessary examinations for patients with a low probability, especially young females, should be reduced which can avoid overdiagnosis.

In clinical practice, however, physicians need to comprehensively consider other clinical factors to avoid delays in diagnosis and treatment. Therefore, decision makers can make personalized decisions based on the characteristics of different populations. This can not only help high-risk patients to be diagnosed and treated as soon as possible, but also make effective use of medical resources. This further emphasizes the importance of individual characteristics, which would be of great significance for the government and policymakers in health-related fields when setting health care policies and for insurance companies when designing insurance programs.

The contribution of this paper is two-fold. First, we extended the POMDP model to a new problem and studied personalized diagnostic decisions of chronic diseases considering individual characteristics of patients. This model can also be extended to other diseases and other medical decisions such as screening and treatment policies. Second, we derived some structural properties, including the optimal diagnosis time for a certain patient with chronic diseases and the belief threshold of diagnostic policies for different populations, and investigated the effect of individual characteristics on properties.

## 7. Conclusions

Diagnostic policies of chronic diseases have become a critical research issue for patients and physicians as well as decision makers in health-related fields, which currently has potential problems such as overdiagnosis and underdiagnosis. We constructed a POMDP model for chronic diseases to optimize diagnostic policies incorporating individual characteristics of patients and derived some structural properties, including the existence of the diagnostic threshold and the optimal diagnosis age.

More specifically, from the patient’s perspective, we determined the optimal diagnosis time for a certain patient based on basic screening results; from the perspective of physicians and the government as well as decision makers, we analyzed optimal diagnostic policies, denoted by the belief threshold for populations with different individual characteristics. Our proposed model maximizes health benefits for patients and achieves the trade-off between underdiagnosis and overdiagnosis from a systematic point of view. Furthermore, the application of this model can improve overall public health QALYs and can be an efficient use of resources, especially where available resources, such as essential medical supplies and well-trained personnel, are constrained.

There are several future research directions of this study. Firstly, we only considered the impact of age and gender on the decision-making of invasive examinations, while other risk factors such as family medical history, smoking, and drinking are related to chronic diseases. Secondly, we assumed 100% adherence rate of patients to diagnosis recommendations in POMDP, and hence the impact of adherence behavior on the diagnostic policies is a potential topic for further exploration. Thirdly, an increasing number of patients prefer to play an active role during the decision-making process in clinical practice, which suggests it is critical to incorporate various risk preferences into the process for individuals or populations. Finally, because this paper focused on diagnostic policies, in terms of extending the analysis another research direction is to comprehensively analyze optimal policies of patients during the entire process including screening and treatment.

## Figures and Tables

**Table 1:** Immediate rewards.

$s_{t}$	$a_{t}$	
	W	G
H	1	1−μ
P	1−α	$1-\mu -\alpha \left(1-p_{3}\right)-\beta p_{3}$
C	1−β	1−β
M	1−γ	1−γ
D	0	0

**Table 2:** Model parameters.

Parameter	Description	Value		Source
$i_{t}$	Annual incidence of CHD	ages 40–60	0.0102	Center for Health Statistics and Information [49]
		ages 60–100	0.0278	
$e_{t}$	Probability of health deterioration	ages 40–60	0.0051	Center for Health Statistics and Information [49]; Yin et al. [50]
		ages 60–100	0.0139	
$d_{1}$	Annual mortality from CHD	0.0012		National Center for Cardiovascular Diseases [51]
$d_{2}$	Annual mortality from other reasons	0.06		National Bureau of Statistics [52]
$p_{1}$	Specificity of basic screening	0.62		Hatzidakis et al. [19]
$p_{2}$	Sensitivity of basic screening	0.74		Hatzidakis et al. [19]
α	Annual loss in state P	0.2 QALYs		Amemiya and Takao [53]
β	Annual loss in state C	0.2 QALYs		Kansara et al. [35]
γ	Annual loss in state M	0.3 QALYs		Gillespie et al. [38]
λ	Discount factor	0.97		Li et al. [26]
μ	Loss associated with a single CAG	0.03 QALYs		Average length of stay per patient
$p_{3}$	Diagnostic rate of CAG	0.41		Clinical data

**Table 3:** Diagnostic rate based on individual characteristics.

Age	Males	Females
40–50	0.38	0.15
51–60	0.45	0.31
61–70	0.52	0.36
71–80	0.67	0.42
81–90	0.78	0.71
91–100	1.00	0.00

**Table 4:** Comparison of QALYs between the optimal policy to other policies.

	$b_{40}\left(P\right)$	Optimal Policy	QALYs under Optimal Policy	Improvements Compared with the Other Policies	Percentage Improvement
Males	0	W	23.6907	0.0300	0.127
	0.5	G	21.6686	0.0383	0.177
	1	G	19.6765	0.1067	0.545
Females	0	W	23.6907	0.0300	0.127
	0.5	W	21.6303	0.0036	0.017
	1	G	19.5927	0.0228	0.116

## Data Availability

The data presented in this study are available on request from the corresponding author.

## Appendix A

To prove the aforementioned propositions, we need to prove related lemmas. First, we present the expected discounted future rewards under two policies after age t for five states H, P, C, M and D, respectively. The rewards can be written as

$${\bar{R}}_{t}\left(H\right)={\bar{r}}_{t}\left(H,W\right)+\lambda \left(p_{t}(H\left|H,W\right.){\bar{R}}_{t+1}\left(H\right)+p_{t}(P\left|H,W\right.){\bar{R}}_{t+1}\left(P\right)\right),$$

$${\bar{R}}_{t}\left(P\right)={\bar{r}}_{t}\left(P,W\right)+\lambda \left(p_{t}(P\left|P,W\right.){\bar{R}}_{t+1}\left(P\right)+p_{t}(M\left|P\right.)R_{t+1}\left(M\right)\right),$$

$$R_{t}\left(H\right)={\bar{r}}_{t}\left(H,G\right)+\lambda \left(p_{t}(H\left|H,G\right.){\bar{R}}_{t+1}\left(H\right)+p_{t}(P\left|H,G\right.){\bar{R}}_{t+1}\left(P\right)\right),$$

$$R_{t}\left(P\right)={\bar{r}}_{t}\left(P,G\right)+\lambda \left(p_{t}(P\left|P,G\right.){\bar{R}}_{t+1}\left(P\right)+p_{t}(C\left|P\right.)R_{t+1}\left(C\right)+p_{t}(M\left|P\right.)R_{t+1}\left(M\right)\right),$$

$$R_{t}\left(C\right)={\bar{r}}_{t}\left(C\right)+\lambda p_{t}(C\left|C\right.)R_{t+1}\left(C\right),$$

$$R_{t}\left(M\right)={\bar{r}}_{t}\left(M\right)+\lambda p_{t}(M\left|M\right.)R_{t+1}\left(M\right),$$

$$R_{t}\left(D\right)={\bar{r}}_{t}\left(D\right).$$

**Lemma** **A1.** ${\bar{R}}_{t}\left(P\right)\geq R_{t}\left(M\right)$ *for all* t∈T.

**Proof.** From the above reward functions and the assumption α≤β≤γ, we have

$$\begin{array}{ll} {\bar{R}}_{t}\left(P\right)-R_{t}\left(M\right) & ={\bar{r}}_{t}\left(P,W\right)+\lambda \left(p_{t}(P\left|P,W\right.){\bar{R}}_{t+1}\left(P\right)+p_{t}(M\left|P,W\right.)R_{t+1}\left(M\right)\right)-\left({\bar{r}}_{t}\left(M\right)+\lambda p_{t}(M\left|M\right.)R_{t+1}\left(M\right)\right)\\ \; & =\gamma -\alpha +\lambda (1-d_{2})(1-e_{t})\left({\bar{R}}_{t+1}\left(P\right)-R_{t+1}\left(M\right)\right)+\lambda (1-d_{2})d_{1}R_{t+1}\left(M\right)\\ \; & \geq \lambda (1-d_{2})(1-e_{t})\left({\bar{R}}_{t+1}\left(P\right)-R_{t+1}\left(M\right)\right). \end{array}$$

When the patient is in state M, $R_{t}\left(M\right)$ is greater than 0, thereby the above inequality holds. Expanding this inequality for t+1,t+2,…,100, we have

$${\bar{R}}_{t}\left(P\right)-R_{t}\left(M\right)\geq \left(\gamma -\alpha \right){\left(\lambda (1-d_{2})\right)}^{100-t}*\prod_{j=t}^{100}\left(1-e_{j}\right)\geq 0,$$

which implies ${\bar{R}}_{t}\left(P\right)\geq R_{t}\left(M\right)$ for all t∈T. Lemma (A1) means that the patient in state P has expected QALYs no less than that in state M given the decision to wait.

**Lemma** **A2.** ${\bar{R}}_{t}\left(H\right)\geq {\bar{R}}_{t}\left(P\right)$ *for all* t∈T.

**Proof.** From the above reward functions, we have

$$\begin{array}{ll} {\bar{R}}_{t}\left(H\right)-{\bar{R}}_{t}\left(P\right) & ={\bar{r}}_{t}\left(H,W\right)+\lambda \left(p_{t}(H\left|H,W\right.){\bar{R}}_{t+1}\left(H\right)+p_{t}(P\left|H,W\right.){\bar{R}}_{t+1}\left(P\right)\right)-\\ \; & \left({\bar{r}}_{t}\left(P,W\right)+\lambda \left(p_{t}(P\left|P,W\right.){\bar{R}}_{t+1}\left(P\right)+p_{t}(M\left|P,W\right.)R_{t+1}\left(M\right)\right)\right)\\ \; & =\alpha +\lambda (1-i_{t})(1-d_{2}){\bar{R}}_{t+1}\left(H\right)+\lambda (1-d_{2}){\bar{R}}_{t+1}\left(P\right)\left(i_{t}-1\right)+\lambda e_{t}(1-d_{2})\left({\bar{R}}_{t+1}\left(P\right)-R_{t+1}\left(M\right)\right)\\ \; & \geq \lambda (1-i_{t})(1-d_{2})\left({\bar{R}}_{t+1}\left(H\right)-{\bar{R}}_{t+1}\left(P\right)\right). \end{array}$$

From Lemma (A1), the above inequality holds. Expanding this inequality for t+1,t+2,…,100, we have

$${\bar{R}}_{t}\left(H\right)-{\bar{R}}_{t}\left(P\right)\geq \alpha {\left(\lambda (1-d_{2})\right)}^{100-t}*\prod_{j=t}^{100}\left(1-i_{j}\right)\geq 0,$$

hence the result holds. Lemma (A2) means that the patient in state H has expected QALYs no less than that in state P given the decision to wait.

**Lemma** **A3.** $R_{t}\left(C\right)\geq {\bar{R}}_{t}\left(P\right)$ *for all*t∈T*when* α=β.

**Proof.** From the above reward functions and the assumption α≤β≤γ, we have

$$\begin{array}{ll} R_{t}\left(C\right)-{\bar{R}}_{t}\left(P\right) & ={\bar{r}}_{t}\left(C\right)+\lambda p_{t}(C\left|C\right.)R_{t+1}\left(C\right)-\left({\bar{r}}_{t}\left(P,W\right)+\lambda \left(p_{t}(P\left|P,W\right.){\bar{R}}_{t+1}\left(P\right)+p_{t}(M\left|P,W\right.)R_{t+1}\left(M\right)\right)\right)\\ \; & =\alpha -\beta +\lambda \left(1-d_{2}\right)\left(R_{t+1}\left(C\right)-{\bar{R}}_{t+1}\left(P\right)+e_{t}\left({\bar{R}}_{t+1}\left(P\right)-R_{t+1}\left(M\right)\right)\right)\\ \; & \geq \alpha -\beta +\lambda \left(1-d_{2}\right)\left(R_{t+1}\left(C\right)-{\bar{R}}_{t+1}\left(P\right)\right). \end{array}$$

From Lemma (A2), the above inequality holds. Expanding this inequality for t+1,t+2,…,100, we have

$$R_{t}\left(C\right)-{\bar{R}}_{t}\left(P\right)\geq \left(\alpha -\beta \right){\left(\lambda (1-d_{2})\right)}^{100-t}\geq 0,$$

hence the result holds when α=β. Lemma (A3) means that the patient in state C has expected QALYs no less than that in state P.

**Lemma** **A4.** $R_{t}\left(C\right)\geq R_{t}\left(M\right)$*for all*t∈T.

**Proof.** From the above reward functions and the assumption α≤β≤γ, we have

$$\begin{array}{ll} R_{t}\left(C\right)-R_{t}\left(M\right) & ={\bar{r}}_{t}\left(C\right)+\lambda p_{t}\left(C\left|C\right.\right)R_{t+1}\left(C\right)-\left({\bar{r}}_{t}\left(M\right)+\lambda p_{t}(M\left|M\right.)R_{t+1}\left(M\right)\right)\\ \; & =\gamma -\beta +\lambda \left(1-d_{2}\right)\left(R_{t+1}\left(C\right)-R_{t+1}\left(M\right)\right)+\lambda \left(1-d_{2}\right)d_{1}R_{t+1}\left(M\right)\\ \; & \geq \lambda \left(1-d_{2}\right)\left(R_{t+1}\left(C\right)-R_{t+1}\left(M\right)\right). \end{array}$$

When the patient is in state M, $R_{t}\left(M\right)$ is greater than 0, thereby the above inequality holds. Expanding this inequality for t+1,t+2,…,100, we have

$$R_{t}\left(C\right)-R_{t}\left(M\right)\geq \left(\gamma -\beta \right){\left(\lambda (1-d_{2})\right)}^{100-t}\geq 0,$$

hence the result holds. Lemma (A4) means that the patient in state C has expected QALYs no less than that in state M.

**Lemma** **A5.** $R_{t}\left(H\right)\geq R_{t}\left(P\right)$ *for all*t∈T.

**Proof.** From the above reward functions and the assumption α≤β≤γ, we have

$$\begin{array}{ll} R_{t}\left(H\right)-R_{t}\left(P\right) & ={\bar{r}}_{t}\left(H,G\right)+\lambda \left(p_{t}\left(H\left|H,G\right.\right){\bar{R}}_{t+1}\left(H\right)+p_{t}\left(P\left|H,G\right.\right){\bar{R}}_{t+1}\left(P\right)\right)\\ \; & -\left({\bar{r}}_{t}\left(P,G\right)+\lambda \left(p_{t}\left(P\left|P,G\right.\right){\bar{R}}_{t+1}\left(P\right)+p_{t}\left(C\left|P\right.\right)R_{t+1}\left(C\right)+p_{t}\left(M\left|P\right.\right)R_{t+1}\left(M\right)\right)\right)\\ \; & =\alpha \left(1-p_{3}\right)+\beta p_{3}+\lambda (1-d_{2})(1-i_{t})\left({\bar{R}}_{t+1}\left(H\right)-{\bar{R}}_{t+1}\left(P\right)\right)\\ \; & +\lambda (1-d_{2})\left(\left(p_{3}+e_{t}\left(1-p_{3}\right)\right){\bar{R}}_{t+1}\left(P\right)-p_{3}R_{t+1}\left(C\right)-e_{t}(1-p_{3})R_{t+1}\left(M\right)\right). \end{array}$$

From Lemmas (A1) and (A3), we have k≥1, where $k=\frac{R_{t+1}\left(C\right)-{\bar{R}}_{t+1}\left(P\right)}{{\bar{R}}_{t+1}\left(P\right)-R_{t+1}\left(M\right)}$. Because the probability of health deterioration in a CAG diagnosis of healthy is not more than k times the probability of a CAG diagnosis of having CHD, we have $p_{3}k\geq e_{t}(1-p_{3})$, and hence the above inequality holds. Expanding this inequality for t+1,t+2,…,100, we have

$$R_{t}\left(H\right)-R_{t}\left(P\right)\geq \alpha {\left(\lambda (1-d_{2})\right)}^{100-t}*\prod_{j=t}^{100}\left(1-i_{j}\right)\geq 0,$$

which implies $R_{t}\left(H\right)\geq R_{t}\left(P\right)$ for all t∈T. Lemma (A5) means that the patient in state H has expected QALYs no less than that in state P given the decision to diagnosis.

**Lemma** **A6.** $R_{t}\left(P\right)-{\bar{R}}_{t}\left(P\right)\geq -\mu$ *for all* t∈T.

**Proof.** From the above reward functions, we have

$$\begin{array}{ll} R_{t}\left(P\right)-{\bar{R}}_{t}\left(P\right) & ={\bar{r}}_{t}\left(P,G\right)+\lambda \left(p_{t}\left(P\left|P,G\right.\right){\bar{R}}_{t+1}\left(P\right)+p_{t}\left(C\left|P\right.\right)R_{t+1}\left(C\right)+p_{t}\left(M\left|P\right.\right)R_{t+1}\left(M\right)\right)\\ \; & -\left({\bar{r}}_{t}\left(P,W\right)+\lambda \left(p_{t}\left(P\left|P,W\right.\right){\bar{R}}_{t+1}\left(P\right)+p_{t}\left(M\left|P\right.\right)R_{t+1}\left(M\right)\right)\right)\\ \; & =-\mu +\left(\alpha -\beta \right)p_{3}+\lambda \left(1-d_{2}\right)\left(p_{3}\left(R_{t+1}\left(C\right)-{\bar{R}}_{t+1}\left(P\right)\right)+e_{t}p_{3}\left({\bar{R}}_{t+1}\left(P\right)-R_{t+1}\left(M\right)\right)\right). \end{array}$$

From Lemmas (A1) and (A3), hence the result holds.

**Proof of Proposition A1.** Due to $R_{t}\left(H\right)-{\bar{R}}_{t}\left(H\right)=-\mu$, we have $\Delta QALYs=b_{t}\left(P\right)\left(R_{t}\left(P\right)-{\bar{R}}_{t}\left(P\right)+\mu \right)-\mu$. For any ∀t∈T, by taking the derivative of ΔQALYs with respect to $b_{t}\left(P\right)$, we have $\frac{d\Delta QALYs}{db_{t}\left(P\right)}\geq 0$ according to Lemma (A6). Then, we have ΔQALYs increases with $b_{t}\left(P\right)$ at each period for any t.

**Proof of Proposition A2.** By Lemma (A5), we have $\frac{dv_{t}\left(b_{t},G\right)}{db_{t}\left(P\right)}=-\left(R_{t}\left(H\right)-R_{t}\left(P\right)\right)\leq 0$. Similarly, we conduct the derivation for $v_{t}\left(b_{t},W\right)$, $\frac{dv_{t}\left(b_{t},W\right)}{db_{t}(P)}=-\left({\bar{R}}_{t}\left(H\right)-{\bar{R}}_{t}\left(P\right)\right)\leq 0$ by Lemma A2. Because $b_{t}\left(P\right)\in \left[0,1\right]$, the value of ΔQALYs is finite in T. As a result, there exists an optimal diagnosis time $t^{*}\in T$ for the patient with the probability of having CHD $b_{t}\left(P\right)$, which maximizes the incremental rewards in QALYs of CHD diagnosis. And the optimal time is uniquely determined by the following equation

$$t^{*}\in \arg\max\left(v_{t}\left(b_{t},G\right)-v_{t}\left(b_{t},W\right)\right)=\arg\max\left(b_{t}\left(P\right)\left(R_{t}\left(P\right)-{\bar{R}}_{t}\left(P\right)+\mu \right)-\mu \right).\;□$$

**Proof of Proposition A3.** According to Lemma (A6), we have $R_{t}\left(P\right)-{\bar{R}}_{t}\left(P\right)+\mu \geq 0$. If $b_{t}\left(P\right)\leq \frac{\mu }{R_{t}\left(P\right)-{\bar{R}}_{t}\left(P\right)+\mu }$, $\Delta QALYs=b_{t}\left(P\right)\left(R_{t}\left(P\right)-{\bar{R}}_{t}\left(P\right)+\mu \right)-\mu \leq 0$ holds, which implies that the optimal diagnosis decision is wait, namely $a_{t}^{*}=W$. In contrast, if $b_{t}\left(P\right)>\frac{\mu }{R_{t}\left(P\right)-{\bar{R}}_{t}\left(P\right)+\mu }$, we have $v_{t}\left(b_{t},G\right)>v_{t}\left(b_{t},W\right)$, i.e., ΔQALYs>0 holds, which implies that the optimal diagnosis decision $a_{t}^{*}=G$. This proposition can be categorized and proved by considering the two different cases presented below. Case 1 ($a_{t}^{*}\left(b_{t}\left(P\right)=1\right)=G$): In this case, $v_{t}\left(b_{t}=1,W\right)<v_{t}\left(b_{t}=1,G\right)$. From Proposition (2), $v_{t}\left(b_{t},G\right)$ and $v_{t}\left(b_{t},W\right)$ are non-increasing in $b_{t}\left(P\right)$. From Lemmas (A1) and (A3), we have $\frac{dv_{t}\left(b_{t},G\right)}{db_{t}\left(P\right)}\geq \frac{dv_{t}\left(b_{t},W\right)}{db_{t}(P)}$. It follows that $v_{t}\left(b_{t},W\right)$ and $v_{t}\left(b_{t},G\right)$ have at most one intersection of $b_{t}^{*}\left(P\right)$. As a result, if $b_{t}\left(P\right)>b_{t}^{*}\left(P\right)$, $a_{t}^{*}\left(b_{t}\right)=G$; if $b_{t}\left(P\right)\leq b_{t}^{*}\left(P\right)$, $a_{t}^{*}\left(b_{t}\right)=W$. Case 2 ($a_{t}^{*}\left(b_{t}\left(P\right)=1\right)=W$): By the condition of this case, ΔQALYs is not greater than 0. From Proposition (1), ΔQALYs is positively correlated with $b_{t}\left(P\right)$. As a result, the optimal diagnosis decision is wait at any $b_{t}\left(P\right)$, which implies $a_{t}^{*}\left(b_{t}\right)=W$, if $b_{t}\left(P\right)\leq b_{t}^{*}\left(P\right)$ and $a_{t}^{*}\left(b_{t}\right)=G$, if $b_{t}\left(P\right)>b_{t}^{*}\left(P\right)$, where $b_{t}^{*}\left(P\right)=1$.
